# Embedding positive psychology into curriculum to promote posttraumatic growth, psychological flexibility, and socio-emotional competencies in higher education

**DOI:** 10.3389/fpsyg.2024.1450192

**Published:** 2024-09-27

**Authors:** Kathleen Chim, JoJo T. C. Lai, Benjamin Tak Yuen Chan

**Affiliations:** Li Ka Shing School of Professional and Continuing Education, Hong Kong Metropolitan University, Hong Kong, China

**Keywords:** embedded curriculum, educational intervention, positive psychology, posttraumatic growth, psychological flexibility, socio-emotional competencies, life transition, higher education

## Abstract

**Background:**

The benefits of positive psychology interventions (PPIs) have become increasingly popular. While there is an emerging evidence base on the effectiveness of applying positive psychology in curriculum as preventive, early mental health intervention for higher education students, little is known about the content and pedagogy in these promising courses.

**Objective:**

This article describes (a) the rationale for and development of a positive psychology course embedded into the curriculum that aims to foster posttraumatic growth, psychological flexibility, and socio-emotional competencies for higher education students; and (b) a mixed-method non-randomised pre-post study to evaluate the effectiveness of the positive psychology course in achieving positive participant outcomes.

**Methods:**

Higher education students from different disciplines will be enrolled to a general education course, “Positive Psychology and Personal Growth”, where they will learn progressive topics and complete summative assessments related to key areas in positive psychology. In addition to lectures, participants will engage in constructivist-based experiential activities that are guided by research on PPIs, life-design interventions and constructivist learning principles. The primary outcome is posttraumatic growth, and the secondary outcomes are psychological flexibility, and socio-emotional competencies.

**Expected results:**

We hypothesised that after the curriculum-embedded PPI, participants will have significantly higher levels of posttraumatic growth, psychological flexibility, and socio-emotional competencies. Results derived from the questionnaire survey will be supported by corroborating evidence identified from qualitative analysis of participants' summative assessments and follow-up semi-structured interviews on their perceptions of the present course.

**Discussion:**

The current study will fill in a gap in existing intervention research and practise in curriculum-embedded PPIs and promote research transparency and pedagogical advancement. The intervention provides guidance and recommendations for educators to consider embedding positive psychology into the formal curriculum as cost-effective, low-intensity, structured, and sustainable educational interventions for higher education students.

## 1 Introduction

Transitioning into higher education can be an exciting, yet stressful experience. The experience is typically characterised by constant changes and multiple stressors as students adjust and learn to become independent adults, autonomous learners, and competitive graduates for jobs and financial challenges (Parker et al., 2004; Anthoney et al., 2017). More importantly, this major life experience occurs during a critical period of psychosocial development, as the onset of lifetime emotional and mental health issues often emerges before age 24 (Kessler et al., 2005). Studies have shown that higher education experience can contribute to the development or exacerbation of existing mental health issues in this population (Duffy et al., 2019; Schmits et al., 2021). The experience was associated with heightened psychological distress and decreased psychological wellbeing (Conley et al., 2020); for some students, distress levels rise in the first year of studies and never return to those recorded before entering higher education (Bewick et al., 2010). The COVID-19 pandemic has further exacerbated mental health issues among higher education students. A recent literature review examining 32 studies revealed increased levels of stress, anxiety, depression, insomnia, obsessive-compulsive disorder, and suicidal ideation in this population (Zarowski et al., 2024). In addition to transition-related stress, research suggests a high prevalence of traumatic life events among this population, ranging from 67% to 84% (Read et al., 2011). A history of trauma significantly increases the risk of mental health issues such as anxiety, depression (Cámara and Calvete, 2021), post-traumatic stress disorder symptoms (Read et al., 2011), and suicidal ideation (Macalli et al., 2020) in higher education students.

### 1.1 Mental health issues and help-seeking behaviour

Student mental wellbeing and learning go hand in hand (Seligman, 2012; Seligman et al., 2009). Poor mental health has been shown to adversely affect academic self-efficacy, progression, persistence, retention, and performance, as well as perceived physical ill health and greater disability (Leow et al., 2024; Verger et al., 2009; Roh et al., 2010). Despite the prevalent and worsening mental health issues in this population, the emotional context of learning is often “*side-lined in higher education*' (Hill et al., 2019, p. 167). In addition, only one third of higher education students were found to have accessed services during their study, with around one fifth of students using outpatient services, as revealed in the first systematic review and meta-analysis of students” use of mental health services (Osborn et al., 2022). A number of common barriers to accessing formal support have been reported in previous studies, such as stigmatising experiences, self-stigma, poor mental health literacy, difficulties and concerns surrounding disclosure, complex and severe mental health issues, complexity and limited availability to approach support services, and a lack of trust in mental health professionals (Broglia et al., 2021; Cage et al., 2020).

Given the dramatic increases in demand for mental health support, limited resources, and the low help-seeking behaviour, higher education institutions can no longer take a “*reactive, individualised approach*” to addressing student wellbeing (Hobbs et al., 2022, p.2). Instead, a proactive, “whole university approach” should be adopted to systematically and holistically enhance their practise and approaches to student mental health and wellbeing through collaborative partnerships and organisational, structural and environmental changes (Hughes and Spanner, 2019; Baik et al., 2017).

### 1.2 Mental health embedded in curriculum

Higher education settings provide diverse learning opportunities which directly impact all students. More specifically, it is argued that every aspect of a student's experience should be designed to support their mental wellbeing, with interventions integrated naturally to help them develop their own self-management skills, resilience, and wellbeing (Hughes et al., 2018; Lister and Allman, 2024; Lister et al., 2021; Dooris and Doherty, 2010; Hughes and Spanner, 2019). One area that has garnered increasing attention in recent years is the potential benefits of integrating wellbeing into the curriculum.

According to Hughes et al. (2018), “*for students, their curriculum and their engagement with academics are their only guaranteed points of contact with their university*” (p. 12). In their comprehensive, research-informed handbook of enhancing student wellbeing for academic educators and leaders, Baik et al. (2017) echoed the idea that academic curriculum is the one structured and consistent element that gives coherence to student experience. Academic teachers, who design and deliver that curriculum, therefore may support student mental wellbeing “*through teaching innovation and the intentional design of learning environments that are psychologically ‘resource-rich' for students*” (p. 11). Increasingly, higher education institutions are under the pressure to develop alternative and innovative solutions to promote mental health on campus. One potential solution is to address student mental health issues as an educational intervention (Hood et al., 2021).

### 1.3 Positive psychology and its potential application in embedded wellbeing learning

Positive psychology has been increasingly applied in education, health, workplace and organisational settings, and coaching and personal development. Positive psychology is known as the new branch of psychology that systematically studies conditions that contribute to the optimal functioning of individuals, groups, and institutions so that a valuable, flourishing and meaningful life is worth living (Seligman and Csikszentmihalyi, 2000). Higher mental wellbeing is considered a protective factor that could lower the occurrence or severity of mental health issues, particularly depression and anxiety (Duckworth et al., 2005). Recent years have seen an increasing interest in research in positive psychology interventions (PPIs) as a complementary strategy in mental health promotion and treatment (Bolier et al., 2013).

Despite a lack of consensus on the precise definition of PPIs, Sin and Lyubomirsky (2009) defined it as “*treatment methods or intentional activities aimed at cultivating positive feelings, positive behaviours, or positive cognitions*” (p. 467). The broad understanding of intervention goal is to enhance wellbeing through mechanisms aligned with positive psychology concepts and models (Carr et al., 2020). Some examples of mechanisms include Seligman's (2002) PERMA theory which promotes the savouring of positive experiences (P), engagement in absorbing skillful activities (E), relationship enhancement (R), the pursuit of meaning and purpose (M), and achievement (A) (Schueller and Parks, 2014). For Peterson and Seligman's (2004) groundbreaking classification of virtues and strengths, these attributes can be cultivated through developing one's character strengths in all six domains—wisdom, courage, humanity, justice, temperance, and transcendence (Niemiec, 2018).

The past two decades have observed a considerable increase in the number of evaluation studies of PPIs. Meta-analyses have provided evidence that PPIs can improve wellbeing for the general population as well as those with a mental or physical illness, with effect sizes of most PPIs ranging from small to medium (Sin and Lyubomirsky, 2009; Bolier et al., 2013; Carr et al., 2020; Hobbs et al., 2022; White et al., 2019; van Agteren et al., 2021). The robust evidence supporting PPIs suggests their potential effectiveness in enhancing student psychological wellbeing when embedded into curricula (Hobbs et al., 2022). In educational contexts, studies that have examined the effectiveness of courses teaching PPIs have predominantly conducted in schools (Seligman et al., 2009), with systematic reviews demonstrating positive outcomes in students' mental health, psychological wellbeing, interpersonal relationships, and academic performance (Durlak et al., 2011; Benoit and Gabola, 2021; Waters, 2011; Tejada-Gallardo et al., 2020; Shankland and Rosset, 2017). In higher education, a growing number of educational interventions are being developed within a positive psychology framework. While research on the benefits of PPI-embedded courses in higher education is a relatively smaller field, a recent systematic review of 27 studies found that 85% of the studies reported positive effects of positive psychology courses on students' psychological wellbeing, life satisfaction, and happiness (Hobbs et al., 2022). These interventions commonly included learning activities that were designed to promote positive emotions and savouring (focusing on internal experiences during an enjoyable activity; the “Three Good Things” exercise), character strengths (identifying personal strengths and applying them in novel ways), gratitude (writing gratitude letters or keeping a gratitude journal), mindfulness practises (mindful listening), altruistic behaviour (deliberate acts of kindness to strangers), forgiveness (writing a letter forgiving an identified person), and emotional skills training (emotional intelligence, awareness and regulation). Nevertheless, the authors argued that a meta-analysis was not possible due to the insufficient data reported in these reviewed studies. There was also a lack of a detailed understanding of students' experiences in these courses which could only be illuminated through using a qualitative approach. Altogether, while there is scant research on PPIs in higher educational settings, emerging evidence suggests that the application and adaptability of PPLs may have broader cultural relevance in non-Western contexts, indicating promising results for their potential universality (Hall et al., 2024; Lambert et al., 2021).

### 1.4 The current study

The promising and unique potential of higher education settings to embed student mental health into curriculum has been recognised through a variety of published frameworks, guidelines, projects, initiatives, and examples in practise across the globe (Baik et al., 2017; Hughes and Spanner, 2019; Lister et al., 2021; Hill et al., 2019; Berry et al., 2024). These collective efforts are extremely important; however, there is scant research about how curriculum-embedded approaches should be implemented (Upsher et al., 2022) with limited evidence of their effectiveness (Baik et al., 2017; Hobbs et al., 2022). As wellbeing courses continue to grow, it becomes increasingly important that we understand the delivery mechanisms of these courses and to evaluate their effectiveness through empirical and systematic research methods.

While existing studies on student mental health largely place an emphasis on mental ill health and psychopathology, the field is undergoing a paradigm shift whereby personal strengths and resources, as well as positive mental health outcome and psychological traits related to resilience have attracted greater interests in recent years (Randhawa et al., 2023). Indeed, in the stress and trauma literature, the evidence increasingly reports positive psychological changes, commonly termed posttraumatic growth (PTG), in individuals who have struggled with major life experiences that overwhelmed their coping resources and shattered their worldview, but experienced substantial positive transformations and embrace new stressful realities to become stronger as a result (Tedeschi and Calhoun, 2004). PTG may be experienced any of the five major domains—(1) changed philosophies and priorities with greater appreciation of life, (2) a sense of increased inner strength, (3) improved interpersonal relationships, (4) openness to new opportunities and life paths, and (5) deeper spiritual beliefs and a greater sense of purpose and meaning in life. Studies have shown that the phenomenon has been frequently experienced by the general population as well as higher education students worldwide (Taku et al., 2007; Brooks et al., 2016; Oshiro et al., 2023), with one study demonstrating the rates as 55% (Yanez et al., 2011). In addition to individual challenging experiences, the global COVID-19 pandemic has been posited as a trauma, inducing widespread distress, pain, and loss (Bridgland et al., 2021). This pervasive impact may contain elements conducive to fostering PTG (Lewis et al., 2022; Chiang-Hanisko et al., 2024). Altogether, examining how a positive psychology course may facilitate PTG and other related protective constructs, specifically, psychological flexibility (PF) and socio-emotional competencies (SEC), may yield valuable insights into how to foster resilience, enhance wellbeing, and improve educational outcomes among higher education students (Kashdan and Rottenberg, 2010; Gloster et al., 2021; Elias et al., 1997; Zhou and Ee, 2012).

Our central hypothesis is that an embedded education intervention incorporating evidence-based positive psychology principles will enhance the interrelated psychological tools of PTG, PF, and SEC among higher education students. Our objective is to examine the pedagogical practises, with the final aim to suggest evidence-based recommendations that inform future pedagogical strategies and educational policies, targeted educational interventions, and mental health services on campus. By fostering supportive and understanding everyday learning environments, participants in this study are expected to experience significantly increased levels of PTG, higher PF, and improved socio-emotional competencies, all of which will help them better cope with the challenges and stressors of work and life in the future

The present study protocol paper therefore aims to contribute to the growing evidence base for embedded approaches to student mental health by describing the design of a credit-bearing positive psychology course which is offered to students across different disciplines in a higher education institution. Importantly, the protocol allows for addressing existing gaps in knowledge, practise and research concerning wellbeing-embedded approaches aimed at enhancing student mental wellbeing in higher education. First, the study will examine mental health from a growth-based perspective that focuses on using positive psychology as an educational intervention tailored to the needs and challenges faced by higher education students to increase PTG, PF, and SEC as positive outcomes. Second, the effectiveness of the positive psychology course will be evaluated using a mixed methods research design to gain valuable insight regarding the underlying mechanisms of PTG and related protective constructs. The research process will explore the complex personal experiences among higher education students who have their own contextual perception, interpretation, and management of life's challenges. Third, by sharing the methodology and design of the described course and research, the study will examine the underlying curriculum processes that impact students' outcomes. The protocol will be a positive step in providing a detailed, evidence-based resource for educators to consider embedding wellbeing in their faculties, programmes, or courses. Research transparency therefore promotes practise transferability, facilitates replicability of findings, and fosters scholarly and pedagogical dialogue, innovation and advancement.

## 2 Methods and design

### 2.1 Study design and setting

The current study employs a mixed-methods, non-randomised pre-post design to evaluate the (i) effectiveness, (ii) process of change, and (iii) feasibility and acceptability of a proposed positive psychology course. This course aims to facilitate posttraumatic growth, psychological flexibility, and socio-emotional competencies among higher education students. The participants will be full-time students enrolled in higher diploma programs at an urban community college in Hong Kong.

Participants will be recruited through an opportunity sampling method, drawing from the pool of students enrolled in the proposed course, “Positive Psychology and Personal Growth.” A priori power analysis was conducted to determine the required sample size, utilising the following parameters: an alpha level (α) of 0.05, a single-group design with 80% statistical power, and an anticipated effect size of 0.3 (Dhand and Khatkar, 2014; Kadam and Bhalerao, 2010). The analysis indicated that a minimum sample of 90 pairs of pre- and post-intervention data would be necessary to detect the specified effect size. To account for potential attrition and ensure an adequate final sample, the target recruitment goal has been set at 100 participants for this intervention study.

Notably, non-randomised studies offer the distinct advantage of capturing data from real-world settings (Patole, 2021), which is particularly valuable in educational research. However, these studies are often challenged by selection bias and the lack of randomisation, which can introduce confounding variables that may skew the results. To address these limitations, the current study established stringent inclusion and exclusion criteria to ensure a homogeneous participant pool (e.g., a consistent educational and socio-demographic background) (Gardner et al., 2020). This approach helps attribute the findings more accurately to the intervention rather than to differences in participant characteristics.

Moreover, the study will employ statistical analyses to adjust for potential confounders such as age, gender, baseline psychological measures, and socio-economic status (Higgins et al., 2013). This adjustment aims to isolate the effect of the intervention from other variables that might influence the outcomes. Comprehensive mixed-method data collection practises will also be implemented to ensure that all relevant variables are measured and recorded accurately. By collecting detailed and comprehensive data, the study can better account for and adjust for potential sources of bias, thereby enhancing the validity of the findings (Sterne et al., 2019).

### 2.2 Participant recruitment and eligibility

Recruitment of participants will occur through in-class promotion at the commencement of the current course. To be considered eligible for participation, students must meet the following criteria: (i) be 17 years of age or older, (ii) be currently enrolled as a full-time student in a higher diploma programme, (iii) have registered for the course as a core or elective subject, and (iv) demonstrate proficiency in the English language. Individuals who self-identify as being in a psychologically vulnerable state, defined as currently receiving psychological treatment, will be excluded from participation.

A Participant Information Sheet will be distributed to all students enrolled in the course, serving as an invitation to participate in this research study. participants will be granted a minimum of 24 h to deliberate their decision to take part. Participation in the study is entirely voluntary. Students who choose not to participate will retain the right to decline involvement without any consequences. This condition is explicitly stated in the Participant Consent Form for those aged 18 and above, or the Parental Consent Form for those aged 17. Critically, non-participation in the research study will not affect a student's eligibility to attend and complete the proposed course.

### 2.3 The intervention

**Figure 1:**
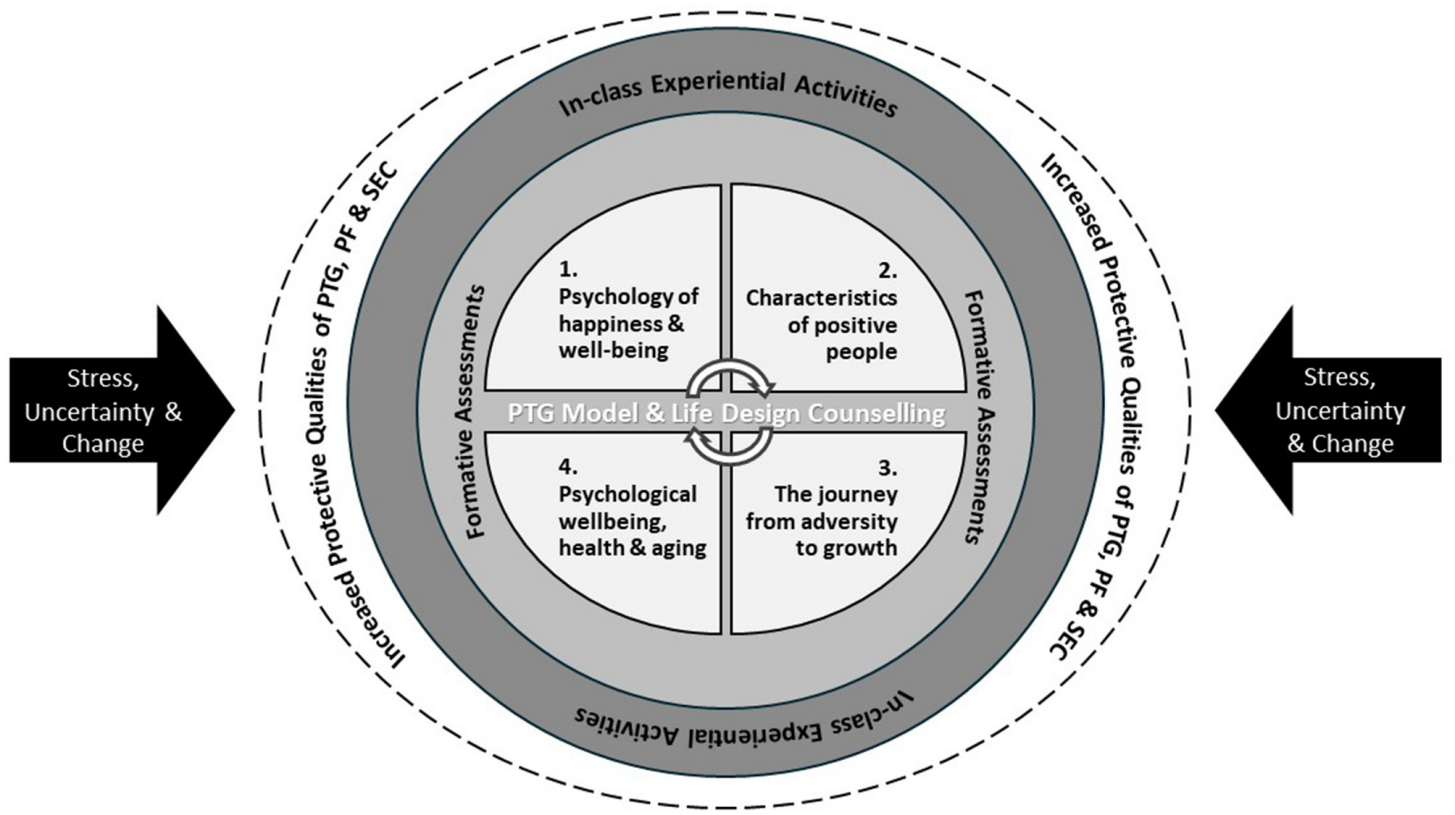
Conceptual summary of course design and pedagogical approaches.

The current course, “Positive Psychology and Personal Growth” adopts a wellbeing-embedded approach centred on an in-depth and progressive exploration of key positive psychology themes. It further incorporates reflective assessments to evaluate learning outcomes and constructivist-based experiential in-class activities to facilitate active learning, knowledge application and transformative personal growth. The curriculum is grounded in the Post-traumatic Growth Model (Tedeschi and Calhoun, 2004; Cann et al., 2010) and the Life-design Counselling Model (Savickas, 2015), as these theoretical frameworks provide a robust foundation for the design, development, and delivery of all course content and materials. The course is explicitly formulated to enhance participants' positive personal resources, namely PTG, PF, and SEC, thereby equipping them with the necessary resources to navigate major life challenges and transitions effectively and promote overall wellbeing. Figure 1 shows a conceptual summary of the design of the current course.

#### 2.3.1 Nurturance of psychological resources to cope with life transitions

To support students in navigating their major life challenges and transitions, the current course aims to help them nurture three inter-related protective psychological tools: posttraumatic growth (PTG), psychological flexibility (PF), and socio-emotional competence (SEC).

Posttraumatic Growth (PTG) refers to the positive psychological changes that arise from grappling with highly challenging life circumstances. It encompasses a deeper appreciation of life, improved relationships, personal strength, new possibilities, and spiritual growth (Tedeschi and Calhoun, 2004). In higher education, PTG enables students to reframe stress and adversity as opportunities for personal development and resilience. This cognitive restructuring enhances problem-solving skills, creativity, and critical thinking (Forgeard, 2013), which are crucial for effectively managing academic and other major life challenges.

Psychological Flexibility (PF) refers to the extent to which an individual “*adapts to fluctuating situational demands, reconfigures mental resources, shifts perspective, and balances competing desires, needs, and life domains*” (Kashdan and Rottenberg, 2010, p. 866). It involves openness to experiences, present-focused awareness, and committed action aligned with personal values, even amid discomfort or adversity (Gloster et al., 2021; Kashdan and Rottenberg, 2010). Among higher education students, PF is related to smoother academic integration and progression (Asikainen, 2018), greater self-efficacy (Jeffords et al., 2020), and diminished negative emotions (Marshall and Brockman, 2016; Ugur et al., 2021).

Socio-Emotional Competence (SEC) encompasses the skills necessary for managing emotions, establishing and maintaining positive relationships, and making responsible decisions. SEC includes self-awareness, self-management, social awareness, relationship skills, and responsible decision-making (Elias et al., 1997; Zhou and Ee, 2012). These competencies are essential for students to acquire the ability to self-monitor their behaviours and self-regulate their learning (Wilson et al., 2001; Zins et al., 2004). They also help students make thoughtful choices about their learning and leverage interpersonal skills to communicate more constructively, enabling them to overcome challenges during problem-solving (Zins et al., 2004), fostering their capacity to adjust to the changing demands in their academic and personal lives (Losada, 2018).

The protective qualities of these constructs, along with their interrelated nature, underpins a robust and purposeful outcome selection of the current course. PTG can foster a mindset that reframes challenges as opportunities for growth, thereby enhancing PF and increasing students' adaptability and resilience (Tedeschi and Calhoun, 2004). Concurrently, PF supports the development of SEC by enhancing students' emotional flexibility, expressivity and management, leading to improved stress-management, more balanced decision-making and better interpersonal relationships (Wersebe et al., 2018; Sarbassova et al., 2024). SEC also provides deliberate reflective tools for the cognitive-restructuring and meaning-making processes that yield PTG (Giordano et al., 2020; Rodríguez and Morales-Rodríguez, 2023). Collectively, these constructs constitute a comprehensive suite of inter-related psychological tools, empowering students to navigate the complexities of higher education and the world of work and thrive in their academic and personal endeavours.

#### 2.3.2 Tailored lecture themes and progressive structure

The Positive Psychology and Personal Growth course is a strength-based module aiming to provide students with an overview of positive psychology, its theoretical underpinnings, and its applications in promoting optimal human functioning, wellbeing, and flourishing (Lomas and Ivtzan, 2016), and specifically, in face of life transitions and adversities. Its intended learning outcomes include (a) Describe key concepts in positive psychology, (b) Analyse the relationships between subjective wellbeing and happiness, (c) Discuss how individuals move from functioning to flourishing, and, (d) Reflect on own meaning of life for enhanced living, personal growth and professional development. They are achieved through introducing students to four tailored lecture themes in a strategic progression to reinforce their psychological resources:

##### 2.3.2.1 Psychology of happiness

Students are introduced to the multidimensional structure of happiness, particularly its hedonic (pleasure-seeking) and eudaimonic (meaning-oriented) aspects (Diener, 2000; Ryan and Deci, 2001; Ryff, 1989). They are guided to learn about Martin Seligman's work on Authentic Happiness and The Good Life with its emphasis on cultivating positive emotions, engagement, relationships, meaning, and accomplishments—collectively known as the PERMA model (Seligman, 2002, 2012). Through this learning, students are encouraged to critically reflect on and actively pursue their unique conception of fulfilling, sustainable happiness. Rather than chasing pleasure, they are guided to align choices and behaviours with core values, fostering personal growth and self-actualization (Ryan et al., 2008). This lecture theme prompts a fundamental shift in perspective, guiding students to move away from externally prescribed notions of success and instead embrace an introspective exploration of personalised pathways to fulfilment (Emery, 2008). It is believed to set the stage for the more profound self-discovery and personal transformation that unfolds throughout the remainder of the curriculum.

##### 2.3.2.2 Characteristics of positive people

The second lecture theme introduces students to a range of psychological strengths and qualities that are characteristic of positive individuals. Emphasis is placed on their development of an authentic self-concept and a strong sense of self-efficacy—the belief in one's capabilities to achieve desired outcomes (Jansen et al., 2015). The application of their character strengths (e.g., gratitude, optimism, curiosity, and perseverance) (Peterson and Seligman, 2004) and mindfulness (i.e., a heightened awareness and acceptance of their immediate experiences) (Shapiro et al., 2006) are further explored. Through this learning, students are encouraged to adopt a growth mindset, viewing setbacks and challenges as opportunities for exercising their positive strengths and qualities, and as platforms for enhancement of adaptive skills (Dweck, 2009; Saraff and Tiwari, 2020). This positive orientation prepares them with psychological resources to re-visit their past adversities, and their increased sense of self-efficacy will serve as a facilitator in the cognitive-restructuring process, which is understood as the “engine” for PTG (Tedeschi and Calhoun, 2004) and will be emphasised in the next lecture theme.

##### 2.3.2.3 The journey from adversity to growth

This lecture theme guides students through the complex process of transforming adversity into personal growth. Beginning with an examination of stress and suffering (Bueno-Gómez, 2017; Fink, 2009), students embark on a journey from distress to coping, adaptation, and the emergence of positive psychological changes. The concept of posttraumatic growth (PTG) is a central focus, highlighting the potential for individuals to undergo positive changes and growth from grappling with challenges (Tedeschi and Calhoun, 2004). By understanding adversity as a catalyst for growth, students are more able to develop a more hopeful and courageous outlook (Di Corrado et al., 2022; Heidarzadeh et al., 2016), fostering a deeper appreciation for their own resilience (Serpa-Barrientos et al., 2023) and the transformative potential inherent in life's challenges.

##### 2.3.2.4 Psychological wellbeing, health, and ageing

Lastly, students are guided through the exploration of existential questions surrounding death and the meaning of life (Baumeister, 1991; Yalom, 1980), such as mortality, life accomplishments, and legacy. This theme ends in the learning of Viktor Frankl's seminal work on logotherapy and the search for meaning (Frankl, 1963), even in the face of profound suffering. Grappling with the inevitability of death can motivate students to live more meaningfully and purposefully through deep reflection on their values and beliefs (Baumeister, 1991). By confronting existential realities, students may be inspired to derive strength and significance from their past experiences, relationships, and life's broader narrative, empowering them to cultivate a more intentional and fulfilling existence (Kenyon, 2000), even amidst profound suffering.

Altogether, the strategic progression of the lecture themes is intentionally designed to guide students through a transformative learning experience. Beyond facilitating comprehension, retention, engagement, and learning motivation, the structure aims to catalyse a fundamental paradigm shift—from a problem-saturated narrative to a growth-oriented perspective (Jakovljević et al., 2012), helping students to develop a deeper appreciation for their inner strengths, resilience, and the inherent potential for personal growth, even in the face of adversity. This paradigm shift can instil a profound sense of courage and hope, equipping students with a more positive, proactive, and adaptive mindset, resulting in greater intentionality and readiness to utilise their personal strengths to overcome current or future life's challenges (Niemiec, 2018).

#### 2.3.3 Strength-fostering formative assessments

A key emphasis of the current course is placed on developing the critical skill of reflecting on life's meaning, as this introspective process facilitates personal growth and deeper learning (Mezirow, 1998). Reflective assessments are identified as the primary mechanism for achieving this growth, particularly in addressing the common tendency among higher education students to engage in self-criticism and focus excessively on their shortcomings (Dweck, 2006).

In the initial reflective essay, students are tasked with describing a challenging event or experience, with the emphasis on their personal perception of difficulty rather than societal norms (Lazarus and Folkman, 1984). They then identify the specific strengths they employed to cope with the challenge, such as courage, creativity, or compassion. Furthermore, students explore the lessons learned from the experience, and explore lessons learned, advice for others, and insights about themselves and their future educational and career decisions (Seligman, 2011). The content of reflective questions integrates ideas and techniques from the PTG workbook (Tedeschi and Moore, 2016) and life-design interventions (Guichard, 2018; Savickas, 2015), providing scaffolding to support students in applying acquired knowledge during their introspective journey (Shepard, 2005). This sequenced approach deepens self-awareness and empowers students to take ownership of their personal growth.

For the second assessment, students revisit their initial essay to reflect on gratitude, considering what they could be thankful for (Emmons and McCullough, 2003). This assessment is grounded in the post-traumatic growth theory which posits that struggling with life's challenges can lead to positive personal development or even transformation over time (Tedeschi and Calhoun, 2004). Students reflect on the potential positive changes they experienced following trauma, such as a greater appreciation for life, new life philosophies, or improved close relationships. They then articulate how these changes have enhanced their overall appreciation for life and for themselves as individuals.

In essence, using reflective writing as a form of formative assessments in this course serve a dual purpose: not only do they evaluate students' acquisition of knowledge, but they also possess a therapeutic capacity to facilitate students' recognition and cultivation of their psychological tools. These latent strengths can function as invaluable resources that students can draw upon throughout the course of their lives (Seligman, 2002).

#### 2.3.4 Constructivist-based experiential in-class activities

According to Dewey's (2001) philosophy of education, education is an unfolding process, and “*every energy should be bent on making the present experience as rich and significant as possible. Then as the present merges insensibly into the future, the future is taken care of* ”. He advocates the use of experience as experimental, reflective and present means to promote students' learning, happiness and growth. Taking this into account, the current course hence utilises a constructivist-experiential pedagogical approach, integrating interactive activities and reflective debriefing sessions that are allied to the tailored lecture themes. The design of these experiential components is guided by research on PPIs (Hobbs et al., 2022; Seligman et al., 2009), life-design interventions (Lewis and Domene, 2021; Savickas, 2005) and constructivist learning principles (Bada and Olusegun, 2015; Hein, 1991; Olsen, 1999), which enable students to actively connect new concepts to their personal backgrounds and lived experiences, fostering deeper comprehension and transformative growth, leading to the enhancement of the selected outcomes, PTG, PF and SEC. Samples of the signature activities are described below.

##### 2.3.4.1 Pursuit of life theme and happiness

This activity guides students through an in-depth self-exploration to uncover the core dimensions that shape their unique life theme (Savickas, 2015, 2016; Stoltz and Barclay, 2015). By reflecting on significant life storeys across different domains, students map their personal psychological landscape. This map illuminates their values, goals, and the distinct version of happiness they innately seek (Emery, 2008).

During the activity, students first thoughtfully document significant storeys related to life-span dimensions like childhood, family, education, work, and relationships, role models, favourite books or movies, and culture etc. In small groups, they vulnerably share their insights and receive supportive feedback. Drawing on these self-discoveries, each student identifies the guiding theme of their developmental journey, and describes its core values, life purposes, and goals. Grounded in this nuanced self-understanding, students then envision a personalised future happiness project, a plan for crafting a life deeply aligned with their authentic self and life theme.

Through this immersive introspective and narrative construction process, students gain profound self-awareness and self-acceptance (Savickas, 2015). They clarify the unique psychological foundations that will guide their pursuit of eudaimonic happiness and continued growth. The group interaction also cultivates deeper interpersonal connexions and perspective-taking skills. Consequently, both the intrapersonal (self-awareness and self-management) and interpersonal (social awareness, relationship skills, and responsible decision-making) dimensions of students' social-emotional competence (SEC) are enhanced (Taylor et al., 2017).

##### 2.3.4.2 Discover my strengths

This activity guides students through a creative visualisation (Gawain, 2016) to connect with a time of passionate engagement, allowing them to uncover their signature strengths. By symbolising energised feelings as an object, then envisioning that object's evolution, students clarify aspirations for their continued growth.

Students are invited to vividly recall feeling enlightened while immersed in an enjoyable activity like studying, working, or a hobby. They re-experience those sensations, then imagine them as a symbolic object, such as s a blazing fire, soaring fireworks, crashing waves, or musical instrument. Next, students contemplate how the object's qualities (e.g., nature, energy, purpose, function) relate to the specific character strengths they utilised during that passionate experience. This symbolic association allows tangible connexion with their unique self (Moser, 2007), particularly, their strengths profile. Lastly, students envision how they'd like their symbolic object, and thus strengths, to evolve over time. Describing the aspirational future state metaphorically represents how they wish to further develop those strengths.

The activity designed to nurture students' sense of ownership over their character strengths. Rather than just learning about strengths, they are allowed to deeply explore how specific strengths empowered them during cherished experiences. Envisioning their strengths' future development further allow students to take charge of their continued growth journey resulting in higher self-continuity and greater subjective wellbeing (Zhang and Chen, 2018).

##### 2.3.4.3 The kintsugi journey

The Kintsugi Journey activity is inspired by the Japanese art of Kintsugi, where broken ceramics are repaired with gold-dusted lacquer and is intended to provide students with a profound experiential metaphor for the human ability to rebuild, heal and grow after adversity (Santini, 2019), representing the concept of PTG (Tedeschi and Calhoun, 2004).

The activity begins by having students decorate their own paper plate with art materials to create a “life vision” plate representing their idealised future. They then exchange their plates with partners and are asked to intentionally damage these plates, symbolising life's disruptions and adversities. Reflecting on the emotional impact of this “destruction,” students are invited to share personal stories of experiencing hardships in their daily lives. This allows them to draw parallels between the activity and their lived experiences integrating challenges into their life narratives. In the final phase, students are invited to rebuild and reassemble their damaged plates, now with visible cracks and imperfections, showcasing their ability to reconstruct their lives and senses of self after adversity, and highlighting the beauty and resilience inherent in their repaired life visions.

On one hand, the plate re-building process can strengthen students' recognition that they could craft lives of profound beauty and meaning by embracing all their experiences—even the most difficult ones (Santini, 2019). On the other hand, as an experiential art activity, it provides a supportive environment for recollection of emotional events (Holt, 2018), reduces anxiety (Mundet-Bolos et al., 2017), encourages open sharing of personal narratives (Margrove, 2015), and facilitates both physical and mental wellbeing in higher education students.

##### 2.3.4.4 End-of-life auction

This activity is designed to guide students to reflect on their personal values and priorities when facing end-of-life and mortality (Breitbart et al., 2004; Vachon et al., 2009). Through an immersive ranking process, students can gain insight into their own belief systems while appreciating diverse viewpoints during this profound life stage. More importantly, this exercise allows students to further consolidate their changed life philosophy and priorities that emerged during the cognitive restructuring explored in the previous lecture theme on PTG. By delving into their deepest thoughts and feelings about mortality, students can solidify their newfound understanding of what truly matters most to them.

In this activity, students imagine themselves as patients nearing the end of life, each with 10,000 “life points” to bid on auction items representing different needs, values, and desires when facing mortality. Items include “Ensuring physical comfort”, “Having a supportive companion”, “Expressing oneself freely”, and “Fulfilling last wishes”. Students can allocate their points based on personal priorities, with the highest bidders declared “winners” for each item. The class then reconvenes to debrief the results, exploring which items received the highest and lowest bids across students. This prompts discussions about common values and priorities when confronting one's own mortality. Students also share how their individual allocations aligned or differed from their peers, reflecting on the insights gained.

This exercise encourages students to confront mortality in a candid yet constructive manner. Although it is also observed that many institutions are ill-equipped to guide students through these profound inquiries, such discussions are indeed important as they support students' sense of meaning and purpose, especially during the crucial developmental stage of emerging adulthood when many are grappling with questions of identity and life direction (van der Meer et al., 2023).

In summary, these constructivist-based experiential activities immerse students in reflective scenarios, facilitating exploration of their authentic selves, embracing life's complexities, and envisioning continued growth amid adversity. This catalyses PTG, PF and SEC through profound personal insight and expanded perspectives. However, it is important to note that positive psychological growth, particularly PTG, is not a guaranteed outcome. Students' progress at their own pace and readiness level—some may experience transformative outcomes, while others require more time or support. Individual differences, contextual factors, and facilitation approach can impact the degree of growth achieved. Though the path is not always linear, these activities create a safe space for students to grapple with existential questions, practise adaptive coping, and nurture essential psychological tools. Additionally, a dual safeguard will be implemented to mitigate any potential discomfort that may arise during the activities.The school counsellors will be informed about the lectures and will be on standby to provide support to students as needed.

Table 1 summarises the lecture themes and key topics, formative assessments and in-class activities of the current course.

**Table 1:** Summary of tailored lecture themes and respective teaching and learning components.

**Lecture theme**	**1. Psychology of happiness**	**2. Characteristics of positive people**	**3. The journey from adversity to growth**	**4. Psychological wellbeing, health and ageing**
Progressive tailored lecture themes that facilitate applications in promoting optimal human functioning, flourishing and life meaning.				
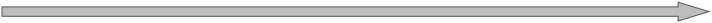				
Key topic	Authentic happiness and the good life	Self-concept, self-esteem and self-efficacy	Stress, distress, adversity and trauma	Death and the meaning of life
	PERMA model	Character-strengths	Recovery and resilience	The pursuit of meaning
	Values, choices and goals	Savouring, mindfulness and flow	Flourishing and posttraumatic growth	Meaning searching and logotherapy
Formative assessment	Assignment 1: personal growth project I - Identify the specific strengths (e.g. courage, creativity, or compassion) employed to cope with a personally challenging event or experience - Explore the lessons learned from the experience and applying the insights on future educational and career decisions		Assignment 2: personal growth project II - Reflect on potential positive psychological changes experienced following the challenging event described in Assignment 1 - Articulate how these changes fall under the topics of PTG and manifest in their daily life stories	
Selected experiential in-class activity and method	Pursuit of life theme and happiness	Discover your strengths	The Kintsugi journey	End-of-life Auction
	a. Identify the guiding developmental theme that underscores one's core values, life purposes, and goals b. Envision a personalised future happiness project that is allied to the authentic self and life theme	a. Visualise an enlightening feeling from an enjoyable activity into an object b. Contemplate on the personal character strengths that the object represents c. Envision how the object i.e., strengths, can evolve over time	a. Build a “life vision” plate representing their idealised future, and re-building it after destruction by peers b. Reflect on the emotional impact of the “destruction”, and the ability to reconstruct their lives and senses of self after adversities	a. Play the role of end-of-life patients to bid on auction items representing different needs, values, and desires when facing mortality b. Reflect on personal priorities and how they indicate personal meanings and purposes of life

#### 2.3.5 Bridging teaching and counselling in classrooms

The curriculum design of the current course employs a cross-disciplinary approach that integrates counselling into teaching. Although traditionally viewed as distinct disciplines, both fields share the common objective of empowering students to become “autonomous with an immense belief in their strength and purpose in life” (Rages, 2022, p. 1136). Consequently, teaching, often perceived as the mere transmission of knowledge, should also encompass the mission of preparing students for life beyond school. This includes equipping them with comprehensive resources (i.e., mental, emotional, social, and strategic) to navigate uncertainty and unpredictability, thereby enhancing their overall wellbeing and life satisfaction (Robinson and Aronica, 2016).

The authors support this paradigm shift in education. By integrating teaching and counselling, the current course intends to provide low-intensity psychological support within the classroom, fostering essential psychological tools alongside the academic curriculum. Students not only learn positive psychology concepts but also internalise them through personal exploration and meaning making, leading to transformative changes towards self-actualization. It is often argued that the capacity of teachers and their workload may hinder the provision of such teacher-led psychological support (Tancinco, 2016). However, as suggested by Rages (2022), this approach functions as an initial phase of counselling and does not require additional time or space outside the classroom. Instead, it emphasises mindfulness, empathy and the acceptance of students' holistic needs, allowing teachers to offer psychological support without overburdening their existing responsibilities.

During the current course, teachers need to shift from the mode of demonstrator or instructor to the mode of a facilitator, drawing upon techniques from low-intensity counselling to guide students through a reflective and meaningful growth journey. They may employ facilitation techniques such as reflective dialogue, character strength-based empowerment, narrative work for change, and emotion-focused meaning-making, which have been suggested in an evidence-based growth career construction psychological intervention (Chim and Lai, 2024). This intervention shares a common emphasis on promoting posttraumatic growth among higher education students. By utilising these techniques, teachers can not only impart knowledge but also touch students' lives by providing them companionship and support, fostering their psychological growth and holistic development.

#### 2.3.6 The implementation

The current course is implemented as a 45 h, one-semester course integrated into the general education curriculum across various academic disciplines. Spanning 13 weeks with 4 h weekly sessions, the course is delivered on-campus as part of the regular coursework. It is compulsory for all enrolled students and is a credit-bearing component with graded assessments contributing to students' overall grade point average (GPA).

While course enrolment and completion are mandatory, participation in the associated research study will be voluntary. Students' rights and ethical considerations related to research involvement are outlined in Section 2.6, ensuring informed consent and protection of participants. This approach allows the intervention to be a core academic experience while respecting individual autonomy regarding research participation.

By integrating the positive psychology curriculum into the general education requirements, the intervention aims to provide a structured and comprehensive learning experience that promotes personal growth, resilience, and wellbeing among the student body, providing widespread exposure to positive psychology principles and practises, fostering a campus culture that prioritises holistic student development across academic disciplines.

### 2.4 Data collection

To assess the intervention's effectiveness, questionnaire data will be gathered from participants both in the first and the final lesson. Furthermore, qualitative data will be obtained through semi-structured interviews with students who have attended the course, the instructors who taught the course, and the programme coordinators who oversaw the implementation process. These interviews will provide experiential insights to complement the quantitative findings. Around 10% of the course participants will be recruited for the interview component of the study.

#### 2.4.1 Outcome questionnaire

To evaluate the intervention's impact on participants, the study will assess changes in their positive personal resources from baseline levels, using the following questionnaires:

##### 2.4.1.1 Post-traumatic growth inventory (PTGI)

Post-traumatic growth (PTG) among participants will be evaluated using the Post-Traumatic Growth Inventory (PTGI), developed by Tedeschi and Calhoun in 1996. This 21-item scale measures the extent to which individuals report positive life changes following a major life crisis or traumatic event. The items assess five domains of PTG, as well as providing a total score. The five domains and their sample items include a sense of increased personal strength (“I have a greater feeling of self-reliance.”), stronger and more meaningful relationships with others (“I more clearly see that I can count on people in times of trouble.”), identification of new possibilities (“I established a new path for my life.”), greater appreciation for life (“I can better appreciate each day.”), and a richer spiritual and existential outlook on life (“I have stringer religious faith.”). Participants rate each item on a scale from 0 (did not experience this change) to 5 (experienced this change to a very great degree). The PTGI has demonstrated good internal consistency (Cronbach's alpha = 0.90) and test-retest reliability (r = 0.71) in previous research (Tedeschi and Calhoun, 1996).

##### 2.4.1.2 Psychological flexibility measure (PsyFlex)

To assess participants' level of psychological flexibility, the study will utilise the 6-item self-report PsyFlex questionnaire developed by Gloster et al. (2021). Sample items include “If need be, I can let unpleasant thoughts and experiences happen without having to get rid of them immediately.” and “I engage thoroughly in things that are important, useful, or meaningful to me.”. This questionnaire is designed to capture state-level psychological flexibility experiences from the past week with high temporal specificity. The items require participants to rate statements on a 5-point scale ranging from 1 (very often) to 5 (very seldom). Total scores can range from 6 to 30, with higher scores indicating greater psychological flexibility. Previous research has demonstrated the questionnaire's excellent psychometric properties, including a one-factor structure, high reliability (r = 0.91; Gloster et al., 2021), and strong validity (Cronbach's alpha = 0.87; Browne et al., 2022).

##### 2.4.1.3 Socio-emotional competencies questionnaire (SECQ)

Participants' socio-emotional competencies will be assessed using the Social-Emotional Competence Questionnaire (SECQ; Zhou and Ee, 2012). It consists of five subscales based on 25 items, including self-awareness, social awareness, self-management, relationship management, and responsible decision-making. The Collaborative for Academic, Social, and Emotional Learning (CASEL) has categorised these competencies into two broader domains: intrapersonal competencies (self-awareness and self-management) and interpersonal competencies (social awareness, relationship skills, and responsible decision-making) (Taylor et al., 2017). Sample items of each subscale are “I understand why I do what I do” (self-awareness), “I understand why people react the way they do” (social awareness), “I stay calm when things go wrong.” (self-management), “I am tolerant of my friend's mistakes” (relationship management), and “When making decisions, I take into account the consequences of my actions” (responsible decision-making). All questions are scored on a 6-point scale ranging from 1 (not at all true of me) to 6 (very true of me). Overall, the authors reported reliability of 0.86 for the SECQ (Zhou and Ee, 2012). Previous research has also demonstrated satisfactory reliabilities for each of the subscales (i.e., self-awareness = 0.64; social awareness = 0.72; self-management = 0.73; relationship management = 0.69; and responsible decision-making = 0.76; Resurrección et al., 2021). Baseline socio-demographic information including age, gender, marital status, religion, education level, work experience, and socioeconomic status will be obtained from participants.

#### 2.4.2 Questionnaire administration

The full set of pre-intervention questionnaires is estimated to require ~30 min for completion, while the post-intervention set should take around 20 min. All measures will utilise the English version, with permissions obtained from the respective authors.

#### 2.4.3 Post-intervention interviews

Upon completion of the proposed course, semi-structured group interviews will be conducted to obtain qualitative feedback from key stakeholders, including participants (students), interventionists (course instructors), and administrators (programme coordinators). The interviews will aim to elicit perspectives on two primary areas: (i) Experiences and perceived processes of change during the course; (ii) Feasibility and acceptability of implementing the proposed course within higher education settings.

An interview guide will be developed a priori to provide a flexible framework for the discussions. This will facilitate a natural conversational flow while ensuring key topics are adequately covered. The guide will also aid in anticipating and preparing for potential challenges in eliciting information on sensitive subject matter.

Each group interview session, including briefing and debriefing, is expected to last ~60 min. The interviews will be conducted remotely via Zoom in Chinese to accommodate participants. All qualitative data will be audio-recorded and subsequently transcribed verbatim to enable in-depth analysis and interpretation of participants' experiences and perspectives.

This multi-stakeholder qualitative evaluation will yield valuable insights into the perceived impact and implementation feasibility of the proposed course, complementing quantitative outcomes to inform future curriculum development and dissemination efforts.

### 2.5 Data analysis

The current study will adopt a mixed-method approach to comprehensively evaluate the efficacy, feasibility, and acceptability of the proposed positive psychology intervention.

#### 2.5.1 Quantitative analysis

Quantitative data will be analysed using IBM SPSS Statistics version 26. Descriptive analyses will be conducted to summarise the socio-demographic characteristics of the sample and investigate participant outcomes following the intervention.

To evaluate the efficacy of the proposed intervention programme, paired-samples *t*-tests will be employed to assess changes in participants' posttraumatic growth, psychological flexibility and socio-emotional competencies from pre- to post-intervention within the same group. A *p* < 0.05 will be considered statistically significant.

#### 2.5.2 Qualitative analysis

Qualitative data obtained from the audio-recorded interviews will undergo thematic analysis adhering to the principles outlined by Bowen (2009) for understanding the process of change happening in the current course (Prochaska et al., 1988), and evaluating the feasibility and acceptability of interventions. The feasibility assessment will encompass various aspects of intervention delivery, including (a) determining the demand for the intervention; (b) evaluating whether implementation proceeded as intended; (c) examining the practicality considering resource and time constraints. The acceptability of the intervention will be examined by exploring how the proposed programme was perceived and received by both the recipients (students) and the interventionists (instructors/administrators).

#### 2.5.3 Mixed methods integration

Adopting a mixed methods design, the quantitative and qualitative findings will be integrated through triangulation to generate a comprehensive understanding. This convergence will yield confirmed findings where both data sources align, discordant findings highlighting divergences, as well as expanded perspectives that extend beyond the individual datasets.

**Figure 2:**
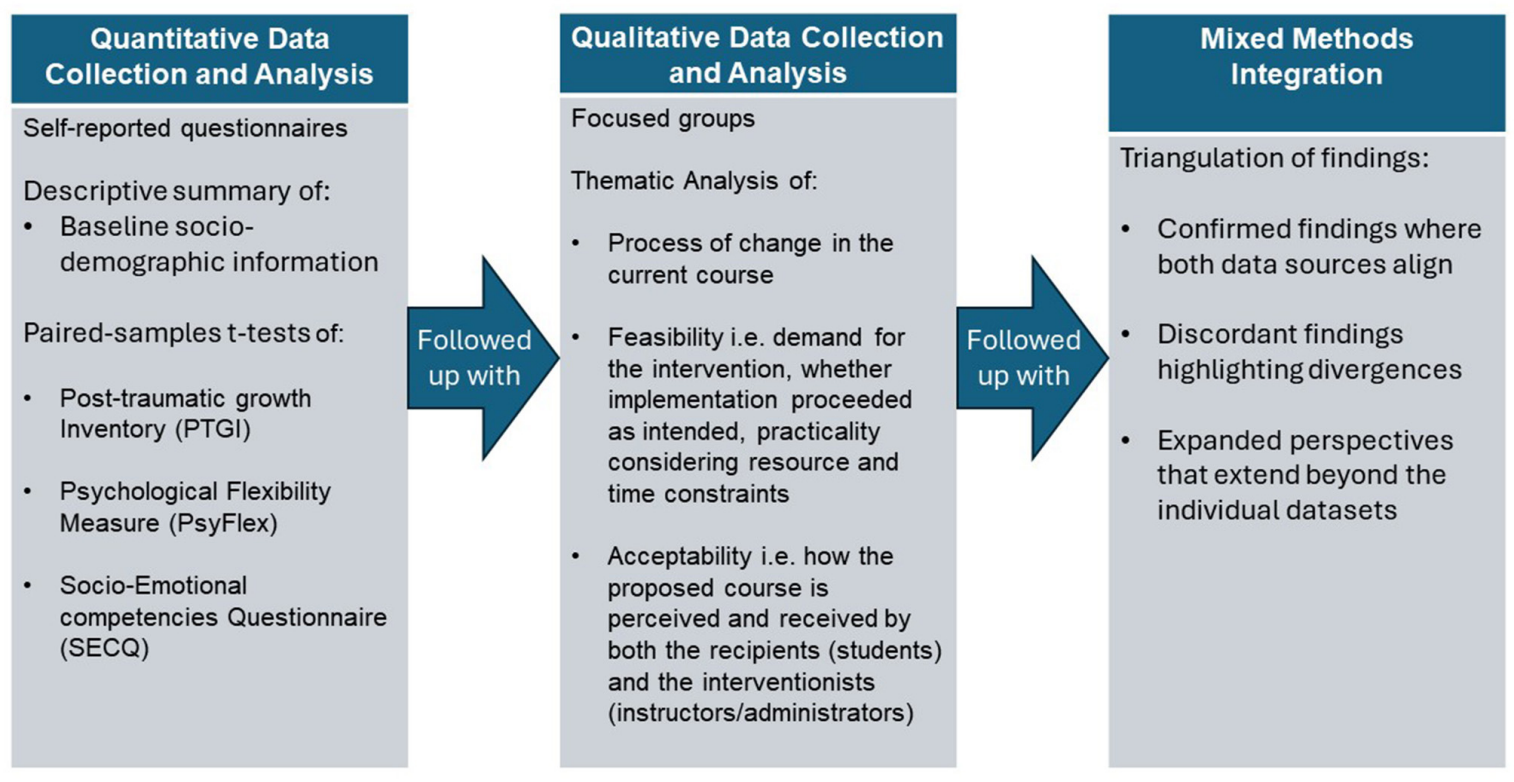
Explanatory sequential mixed method study design.

The combined quantitative and qualitative analyses will provide a multifaceted evaluation of the intervention's efficacy, feasibility, and acceptability, informing future refinements and implementation efforts, guiding educators in designing holistic, growth-based support for higher education students. If effective, the proposed course could provide rationale for implementing PPIs, inform curriculum redesign, and improve student learning and wellbeing. Elements may be adaptable to non-psychology courses, potentially impacting broader educational practises. Effect sizes of the obtained results could help to quantify the strength of the observed pre- and post-intervention changes of the outcome variables, allowing researchers to assess the practical significance of the findings. Figure 2 shows a summary of the explanatory sequential mixed methods study design employed in the current study (Ivankova et al., 2006).

### 2.6 Ethical issues and dissemination

This study adheres to stringent ethical protocols approved by the Hong Kong Metropolitan University Research Ethics Committee (HE-SOL2022/02). All participants will provide informed written consent prior to data collection, underscoring the voluntary nature of their involvement.

While minimal risks are anticipated, the researchers acknowledge the potential for psychological discomfort when participants reflect on challenging experiences. Comprehensive participant information sheets outline these foreseeable risks and provide contact details for local psychological support services. Individuals self-identifying as psychologically unstable will be advised against participation to mitigate potential harm.

Robust measures safeguard participant anonymity and withdrawal rights. Participants will be granted full anonymity, meaning that their identities will not be linked to their data in any way that could identify them. This will be ensured by not collecting any personally identifying information (e.g., names, phone numbers, email addresses) during the data collection process. Their right to withdraw from the study without penalty will be explicitly stated in information sheets and reiterated during data collection phases. Confidentiality is paramount, with access to data restricted to the research team per university guidelines.

Participant data will be securely managed through coded identifiers known only to the principal investigator. Hard copies will be stored in a locked cabinet and digital data will be encrypted and stored on password-protected devices to prevent unauthorised access. Published data will undergo de-identification to protect participant anonymity, ensuring that no individual can be identified from the published results. These rigorous protocols ensure confidentiality and adherence to ethical data management standards.

## 3 Discussion

The higher education sector is experiencing a paradigm shift, moving from a focus on risk mitigation to an emphasis on personal empowerment and growth in response to the widespread and worsening issue of student mental health. Although wellbeing embedded initiatives are gaining attention in higher education as a promising strategy that benefits student's learning and wellbeing, little is known about their pedagogical implementation and empirical evaluation. We expect that the positive psychology course outlined here to possess significant pedagogical value that will inform future educational practises and policies. By integrating evidence-based psychological principles into the curriculum, higher education students can become better equipped with interrelated, protective psychological tools that empower them to navigate and manage life's challenges with greater efficacy and confidence. In the wider context, the importance of SEC is being increasingly recognised to not only play a relevant role in wellbeing, but also other positive aspects such as motivation, academic performance, optimism, life satisfaction, all of which are essential skills for career success (Elmi, 2021; Kim, 2021; Kwok and Gu, 2016; Smith, 2020; Gandía-Carbonell et al., 2022).

The evidence obtained from the described positive psychology course, which will be led by educators with a social sciences background, will help to address the overburdened and under-resourced state of higher education institutions in addressing the rising prevalence of mental health concerns among students. Embedded education intervention incorporating evidence-based positive psychology principles is a preventive, low-cost, sustainable mode of delivery to reach larger numbers of students. With the proliferation of tools and resources aimed at addressing students' mental health in higher education, the current challenge lies in enhancing the capabilities of academic educators to develop teaching and learning practises and create learning environments that provide better support for student mental wellbeing (Baik et al., 2017). The focus on pedagogical implementation in this study therefore provides guidance for educators to creatively design or re-design growth-based educational interventions that offer holistic, seamless support responding to the unique needs of higher education students. Notably, the course offers a safe and guided environment for students to explore often neglected or avoided personal matters, such as values, altruism, gratitude, life adversities, post-traumatic growth, death, and life meaning (Wong, 2011). This open exploration of internal, personal matters is particularly significant in cultures where such matters are typically kept within the self and the family, as they may be perceived as harmful to one's self-image and dignity (Markus and Kitayama, 1991; Uchida and Ogihara, 2012).

Altogether, as a promising field, PPIs embdedded in higher education curricula require more systematic research to evaluate their implementation, application, and effectiveness. Future studies on PPI-embedded educational interventions should explore the implementation fidelity—the degree to which an intervention is delivered as intended—to increase the likelihood of achieving intended longer-term effects (Horowitz et al., 2018). Implementation fidelity consists of six dimensions: programme differentiation (how much the intervention can be differentiated from other programmes), dosage (the intensity of intervention), adherence (the extent to which the intervention content was followed), quality of delivery (whether the intervention key points are easy to process and follow), student responsiveness (participants' level of attention and productivity), and fidelity of receipt (the intervention impact on participants' thinking and behaviour).

## 4 Limitations

This study has several limitations. First, the positive psychology course will be conducted in person. Recently, attention has been paid to the potential positive effects of positive psychology courses conducted online, which therefore enhance accessibility and extend benefits to a broader audience when compared to the traditional face-to-face courses (Dhawan, 2020; Smith et al., 2023; Shimer, 2018). Nevertheless, the course materials described here are readily adaptable for remote teaching and learning based on experiences during the COVID-19 pandemic, facilitating future research to evaluate their effectiveness in distance learning environments. Second, as a non-randomised study without a control group, we are unable to establish causality due to potential confounding variables and the internal validity of the findings may be undermined. Future research should incorporate randomaised controlled trials to enhance causal inference and include control groups to provide comparative data, thereby improving the robustness and generalisability of the findings. Third, to address the limitations of the cross-sectional design of the current study, future research may adopt a longitudinal research design to provide insights into temporal patterns, causal mechanisms, and the potential long-term benefits of wellbeing embedded courses designed within the positive psychology framework. Fourth, there are inherently personal biases in the use of self-reported questionnaires which may potentially affect the validity and reliability of the data collected. The researchers will ensure anonymity, confidentiality, and informed consent, and the use of neutral wording when introducing the project before data collection to minimise biases such as social desirability and acquiescence. Lastly, given that the proposed study will be conducted in the Chinese culture, the generalisability of findings across different populations will be limited. Future research may improve generalisability through cross-cultural replication studies.

## 5 Conclusion

This study aimed to add knowledge to wellbeing-embedded approaches by demonstrating detailed pedagogical strategies and evaluation methods of a positive psychology course designed as a targeted educational intervention to address learning and mental wellbeing among higher education students across disciplines. If proven effective, the educational intervention will provide a rationale for offering PPI as an educational intervention to higher education students. Implementing PPIs in higher education formal learning requires high levels of commitment from faculty and administrator to put into place. Educational policies and senior management strategies can help facilitate this process by providing the necessary framework and support for integrative and interdisciplinary implementation and practises. These aim to contribute to improved educational outcomes, ensuring that students are more comprehensively equipped to embrace future challenges and the complexities of the modern world. Recommendations may include providing professional development training across new and existing faculty members. This training can facilitate the sharing of common knowledge and skills, thereby serving as the foundation for developing a cohesive approach to implementing PPIs in classroom teaching. Institutional funding and support such as grants, partnerships between schools and with external organisations may be put in place to encourage PPI initiatives. Dedicated staff can be nominated as PPI advocates from different departments, disciplines, schools and units to facilitate regular communication and oversee the implementation of PPIs that align with the institution's broader educational goals. The tools provided in this study may guide educators in designing or re-designing curricula, pedagogies, and assessment methods to enhance student learning and wellbeing. Individual elements of the protocol may also be creatively adopted and applied to non-psychology courses, and the modified versions can be evaluated for their potential effectiveness. Developing and disseminating materials and tools, such as step-by-step instructions, case studies, tips, and exercises that support PPI activities on the intranet can enhance availability, promote best practise sharing among staff, and foster a collaborative and supportive teaching and learning environment.

If the educational intervention is not effective, student support service can also be provided to the students to help them manage academic, transition-related, and developmental stressors. At the same time, it can accumulate experience for the further improvement of future positive psychology-focused educational interventions and provide an empirical basis for future development, implementation, and evaluation.

## Data Availability

The original contributions presented in the study are included in the article/supplementary material, further inquiries can be directed to the corresponding author.
